# Chemometric Valorization of Strawberry (*Fragaria x ananassa* Duch.) cv. ‘Albion’ for the Production of Functional Juice: The Impact of Physicochemical, Toxicological, Sensory, and Bioactive Value

**DOI:** 10.3390/foods11050640

**Published:** 2022-02-23

**Authors:** Anica Bebek Markovinović, Predrag Putnik, Boris Duralija, Adela Krivohlavek, Martina Ivešić, Ivana Mandić Andačić, Iva Palac Bešlić, Branimir Pavlić, Jose Manuel Lorenzo, Danijela Bursać Kovačević

**Affiliations:** 1 Faculty of Food Technology and Biotechnology, University of Zagreb, Pierottijeva 6, 10000 Zagreb, Croatia; abebekmarkovinovic@pbf.hr; 2 Department of Food Technology, University North, Trg dr. Žarka Dolinara 1, 48000 Koprivnica, Croatia; 3 Department of Pomology, Division of Horticulture and Landscape Architecture, Faculty of Agriculture, University of Zagreb, Svetošimunska cesta 25, 10000 Zagreb, Croatia; bduralija@agr.hr; 4 Andrija Štampar Teaching Institute of Public Health, Mirogojska 16, 10000 Zagreb, Croatia; adela.krivohlavek@stampar.hr (A.K.); martina.ivesic@stampar.hr (M.I.); ivana.mandicandacic@stampar.hr (I.M.A.); iva.palacbeslic@stampar.hr (I.P.B.); 5 Faculty of Technology, University of Novi Sad, Blvd. Cara Lazara 1, 21000 Novi Sad, Serbia; bpavlic@uns.ac.rs; 6 Centro Tecnológico de la Carne de Galicia, Adva. Galicia n° 4, Parque Tecnológico de Galicia, San Cibrao das Viñas, 32900 Ourense, Spain; jmlorenzo@ceteca.net; 7 Universidade de Vigo, Area de Tecnoloxia dos Alimentos, Facultad de Ciencias de Ourense, 32004 Ourense, Spain

**Keywords:** strawberry, ripeness, functional juice, bioactive compounds, sensory, toxicology

## Abstract

Strawberries (*Fragaria x ananassa* Duch. cv. ‘Albion’) were harvested at two stages of ripeness (75% vs. 100%) and their physicochemical, sensory, toxicological, and bioactive properties were evaluated before and after processing into juice. The fresh fruits and their by-products were also evaluated. During processing into juice, the color change was higher in the fully ripe fruits, confirming the encouraging prospects for using the less ripe strawberries for processing. The analysis of heavy metals (Cu, Zn, Ni, As, Cd, Pb) was carried out, and in juice and by-product samples of 100% maturity, only Pb was higher than the MDK. Of the 566 pesticides analyzed, only cyprodinil was found in the by-products of the strawberries at 75% maturity, while pyrimethanil was detected in all samples. Fresh strawberries of both ripeness levels were rated similarly to the corresponding juices for all sensory attributes studied, indicating that sensory perception was not affected by processing. However, ripeness was found to be an important factor influencing most sensory attributes. The by-products were the materials with the highest levels of all bioactive compounds. Considering all quality parameters evaluated, the chemometric evaluation confirms the suitability of 75% ripe strawberries for processing into functional juice, which could be important for the juice industry.

## 1. Introduction

Strawberries (*Fragaria x ananassa* Duch.) are a very popular fruit among consumers, either as fresh produce for consumption or for processing, e.g., into juices. This raw material is well-aligned with the growing demand for functional foods in the market as consumers increasingly choose products of exceptional quality with added value [1]. Particular attention is being paid to functional products such as strawberry juice, as the strawberry has been shown to be a fruit with numerous health benefits, e.g., anti-inflammatory, anticancer, antioxidant, antidiabetic, antimicrobial, cardioprotective, and neuroprotective effects [2,3]. In addition, the by-product of the strawberry that remains after the fruit is processed into juice has been found to be an excellent source of bioactive compounds with potent antioxidant potential [4,5]. When the strawberry fruit is processed into juice, there is a significant loss of phenolic compounds [6,7]. Most phenolic compounds are contained in the achenes and the receptacles of strawberry fruits, and when strawberry fruits are processed into juice, the phenolic compounds remain bound to the cell wall material. Therefore, most phenolic compounds, including anthocyanins, are better preserved in the by-product than in the juice [8,9,10]. These bioactive compounds, including phenolic acids, flavonoids (such as anthocyanins and flavonols), and tannins are associated with the above-mentioned beneficial effects, especially due to their antioxidant activity [11]; therefore, the great potential of strawberries lies in the production of functional foods. 

On the other hand, strawberries are very fragile fruits, with a short storage time after harvest due to their high respiration rate, and their quality can be affected very quickly, either by handling, storage, or transport, resulting in a short shelf life and high economic losses. Fresh fruits with lower quality are not suitable for processing [12]. Therefore, it is necessary to select cultivars that are more resistant to quality changes during storage and processing. In a recent study, multivariate analysis was successfully applied to select strawberry cultivars suitable for fresh consumption and/or processing. The importance of selecting the appropriate cultivar for the intended purpose was emphasized [13,14]; therefore, this chemometric approach could be an advanced tool for potential industrial purposes [15].

In addition to the genotype, it has been found that growing location, cultivation method and ripening stages have a significant effect on the physicochemical and phytochemical parameters of strawberries [16,17,18]. Sugar content increases and citric and malic acid content decreases with maturity, which contributes to the sweetness of strawberries, making the sugar-acid ratio an important indicator of fruit quality [19,20]. Moreover, the effects of ripening (e.g., almost ripe, partially red; ripe, red; and fully ripe, dark red) have a strong influence on the type and concentration of individual and total polyphenolic compounds [17,21]. Interestingly, the total antioxidant capacity (TAC) of strawberries on the day of harvest was higher in ripe fruits than in unripe fruits, and with increasing storage time, TAC tended to increase in unripe fruits [22]. Ellagic acid content was found to be the highest in unripe fruits and gradually decreased with increasing ripeness [22]. Although totally unripe strawberries are not processed, they could be used to produce juices due to their higher firmness and biological potential, which could form the basis of functional foods. Besides, fully immature strawberries have a firmer texture and are less sensitive to prolonged storage and transportation [23], so they could be a good raw material for the production of functional foods.

Sensory characteristics are extremely important to consumers, and the color of fresh strawberries, as well as strawberry products, is one of the most important quality indicators that consumers look for first [24]. The overall sensory appearance of strawberries is highly dependent on the visual freshness and shininess of the fruit, most likely because shininess decreases as the strawberries dry out and become wrinkled. Perception of glossiness and visual freshness were found to be negatively correlated with color intensity, with bright orange-red strawberry fruit perceived as fresher than darker, more purple-red strawberry fruit [25]. The overall quality of fresh strawberries is influenced by the intensity of red color and the sensation of sweetness, strawberry flavor and overall flavor, fruit aroma, strawberry aroma and overall aroma, while acidity has no effect on the evaluation of overall quality [19,25,26]. The stage of ripeness significantly affects the sensory quality of strawberries. Unripe strawberries have more acidic, citrus, and green flavor attributes than ripe strawberries due to a high concentration of acids, such as citric acid. A study of six strawberry cultivars at immature and ideal ripening stages revealed that the cultivar ‘Albion’ has favorable sensory attributes at both ripening stages, making it an excellent cultivar for both fresh consumption and processing [20]. 

When strawberries are considered from a food safety perspective, the monitoring of pesticide and heavy metal residues should be increased to fully protect consumer health. Heavy metals as naturally occurring elements found throughout the earth’s crust are among the largest contaminants in the food supply and are considered the most serious problem facing our environment [27]. They are not biodegradable or thermally degradable and enter the human body through food; thus, they can accumulate in various body organs, leading to undesirable side effects [28]. It was found that among fruits and vegetables for retail sale, strawberries are the fruits with the highest pesticide content [29]. High pesticide residues in fruits are a sign of the ubiquitous and intensive use of pesticides in their production and distribution. However, there is ample evidence that pesticides pose a potential risk to humans and other living organisms and may also have negative effects on the environment [30]. Therefore, the safety and health benefits of strawberries should be verified before their use in the production of functional foods [31]. Consequently, the aim of this study was to investigate the suitability of the strawberry cultivar ‘Albion’ for the production of functional juice by using chemometrics in quality assessment. For this purpose, the influences of maturity stages, physicochemical properties, toxicology, sensory properties, and bioactive potential were considered. 

## 2. Materials and Methods

### 2.1. Chemicals and Standards

Formic acid (98%, p.a.), sodium carbonate, anhydrous (99.5–100.5%), and hydrochloric acid (37%, *w*/*w*) were purchased from Lach-Ner (Neratovice, Czech Republic). HPLC grade 99% methanol was purchased from Honeywell (Paris, France). Folin-Ciocalteu reagent was purchased from Fisher Scientific UK (Loughborough, UK). Ethanol (96% pure) was purchased from Gram-Mol (Zagreb, Croatia). Gallic acid standard (97.5–102.5%) and quercetin standard (95%) were obtained from Sigma-Aldrich (St. Louis, MO, USA) and Acros Organics (Guangzhou, China), respectively. Chlorogenic acid reference standard (min. 95%), potassium chloride (99.0–100.5%) and sodium acetate anhydride (99%) were purchased from Thermo Fisher (Kandel, Germany). 

### 2.2. Material

The plants were grown in the greenhouse of the private company Jagodar-HB in Donja Lomnica, Zagreb County, (Croatia), in 2021. The growing system was soilless, using plastic bags with coconut coir. Green container plants of the cultivar ‘Albion’ were planted at a density of 10 plants m^–2^ starting in November of the year before harvest. All plants received standard nutrition; the electrical conductivity of the drainage solution was less than 2 dS m^–1^, and pH value in the root zone was between 5.5 and 6.5 during growth and harvest.

**Figure 1:**
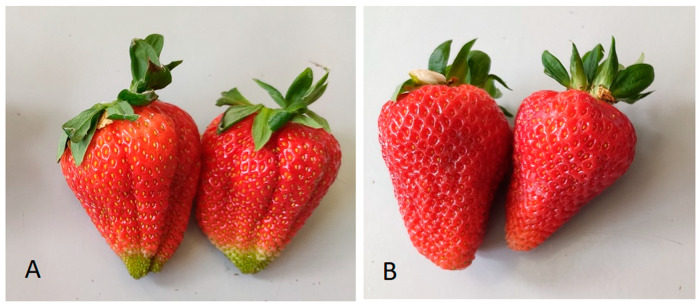
Strawberry fruit (*Fragaria x ananassa* Duch. cv. ‘Albion’) harvested at the stage of technological ripeness (**A**) and at the stage of full ripeness (**B**).

The fruits were harvested in the early morning on 24 May 2021 at different stages of maturity: (i) technological ripening stage (ripe green fruits, about 25% green), (F1), and (ii) full ripening stage (full red, 100% ripeness), (F2), (Figure 1A,B). 

**Figure 2:**
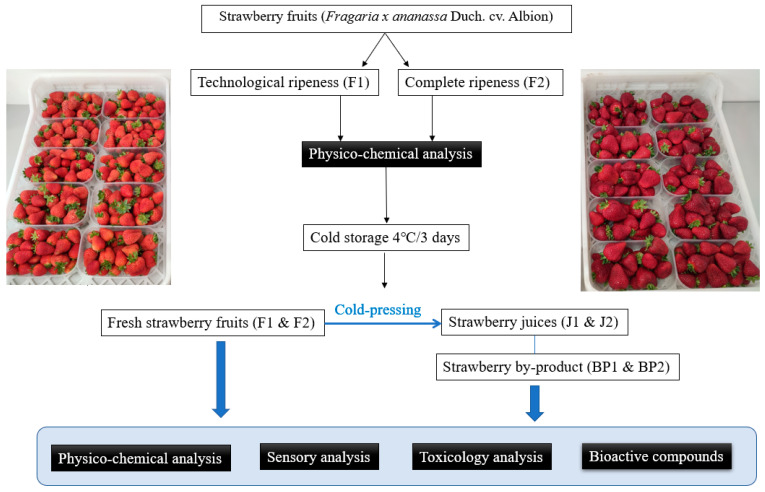
Schematics of the experiment.

Immediately after harvesting, the fruit samples (5 kg for each stage of ripeness) were delivered to the laboratory where the physicochemical analyses were carried out. The general organization of the entire experiment is shown in Figure 2. The fresh fruits were stored at 4 °C for 3 days to test their suitability for processing after a short storage period. Then, physicochemical, sensory, toxicological, and bioactive analyses were performed. At the end of storage, strawberry samples from both ripening stages were processed into juice (J1 and J2) using cold pressing (Kuvings B6000 Slow Juicer, VerVita d.o.o., Zagreb, Croatia), and strawberry by-products BP1 and BP2 were also prepared and analyzed.

### 2.3. Methods

#### 2.3.1. Juice Production Yield 

Strawberry juices (J1 and J2) and their by-products (BP1 and BP2) were prepared by cold pressing the corresponding fresh fruits (F1 and F2). The fresh strawberries (F1 and F2) were weighed before and after removing the calyces, and the removed calyces were weighed separately. The juices obtained (J1 and J2) and their by-products (BP1 and BP2) were also weighed. The juice yield (%) was calculated based on the total weight of the fresh fruits with (Equation (1)) and without calyces (Equation (2)).

(1) $$\mathrm{Juice}\;\mathrm{yield},\%\;=\frac{\mathrm{weight}\;\mathrm{of}\;\mathrm{fruits}\;\mathrm{with}\;\mathrm{calyces}\;\left(g\right)}{\mathrm{weight}\;\mathrm{of}\;\mathrm{juice}\;\left(g\right)}\times 100$$

(2) $$\mathrm{Juice}\;\mathrm{yield},\%\;=\frac{\mathrm{weight}\;\mathrm{of}\;\mathrm{fruits}\;\mathrm{with}\;\mathrm{calyces}\;\left(g\right)-\mathrm{weight}\;\mathrm{of}\;\mathrm{calyces}\left(g\right)}{\mathrm{weight}\;\mathrm{of}\;\mathrm{juice}\;\left(g\right)}\times 100$$

#### 2.3.2. Physicochemical Analysis

Physicochemical analysis for the fresh fruits (F1 and F2) includes the determination of the fruit weight (g), hardness (kg cm^−2^), colorimetric evaluation (CIEL*a*b*), pH, soluble solids content (Brix%) and total acidity (%). In the juice (J1 and J2) and by-product (BP1 and BP2) samples, colorimetric evaluation (CIEL*a*b*), pH, and soluble solids content (Brix%) were determined. 

To determine the fruit weight, color, and firmness at each ripening stage, 10 individual fruits were evaluated. The same fruits were then pureed, homogenized, and filtrated before being used to determine the total soluble solids (TSS), titratable acidity (TA), and pH. The prepared juices (J1 and J2) were also analyzed for color, TSS and pH. The weight of the fruits was determined using an OHAUS AX2202 Adventurer^®^ Precision analytical balance (Ohaus Corporation, Parsippany, NJ, USA).

The color of the fruits and juices was measured using a ColorTec-PCM colorimeter (ColorTec Associates, Clinton, NJ, USA) and expressed as CIEL*a*b* values. Color is defined in three-dimensions using the notation L, a, b. The L* axis represents the lightness of the color (the lower the value, the darker the color). The a* axis represents the balance between red (positive values) and green (negative values) and the b* axis, the balance between yellow (positive values) and blue (negative values). These coordinates allow access to new indices, color change (ΔE*), (Equation (3), chroma (C*), (Equation (4), and hue (H*), (Equation (5)) calculated from:

(3) $$\Delta E^{*}=\sqrt[{}]{\Delta L^{*2\;}+\Delta a^{*2\;}+\Delta b^{*2}}$$

(4) $$C^{*}=\sqrt[{}]{a^{*2\;}+{\;b}^{*2\;}}$$

(5) $$H^{*}={\;\tan}^{-1}\;\left(\frac{b^{*}}{a^{*}}\right)$$

Firmness was measured using a PCE-FM 200 penetrometer (PCE Instruments, Germany) with a 6 mm probe. The firmness value for each fruit was the average of two measurements taken on opposite sides of the fruit in the equatorial fruit zone and was expressed in kg cm^−2^. 

The pulp of each fruit and its juices were used to determine TSS (Brix%) using a digital refractometer (ATAGO Pal-3, ATAGO Co., Tokyo, Japan), while the pH of both samples was determined using a Testo 205 manual pH meter (Testo AG, Lenzkirch, Germany) [32]. The pH meter was calibrated with commercial buffer solutions at pH 7.0 and 4.0. 

The titratable acidity was measured by titration with 0.1 M NaOH using phenolphthalein as indicator and expressed as percentage of citric acid [32].

#### 2.3.3. Toxicology Analysis 

All methods used in this paper for the determination of the toxicological parameters were methods accredited in the flexible scope of accreditation in the Andrija Štampar Teaching Institute of Public Health, Department for Environmental Protection and Health Ecology (accredited since 2003), the National Reference Laboratory for Pesticides in Foods of Plant Origin, for Pesticides in Fruits and Vegetables, Cereals, and the Testing of Pesticides by Individual Methods. For metals, the internal method was used, and for pesticides, the standard method for evaluating foods of plant origin was used. The determination of pesticide residues was carried out using GC-MS and/or LC-MS/MS following acetonitrile extraction/partitioning and cleanup using the dispersive SPE–QuEChERS EN 15662-2018 method.

##### Chemicals

A certified reference material (pesticide mixture) for GC-MS/MS and LC-MS/MS analyses (purity above 99%) was purchased from LabStandards (Budapest, Hungary). The concentrations of each pesticide in CRM are 100 μg mL^−1^. A stock solution was prepared by diluting the CRM 100-fold in acetonitrile to obtain a concentration solution of 1 μg mL^−1^ in 10 mL of acetonitrile. The solution was prepared, and the original mixture of pesticides was stored at a temperature of −20 °C (±2 °C). The stock solution was used to check the recovery of the sample preparation process by spiking samples before extraction. Working solutions of the standard were prepared by the appropriate dilution in a matrix, i.e., acetonitrile extract of strawberry sample. Acetonitrile is 99% pure (pesticide residue grade) and is manufactured by J.T. Baker. QuEChERS salts for extraction and purification of the samples were obtained from Restek Corporation (Bellefonte, PA, USA). QuEChERS salt mixture for sample extraction contains 4 g MgSO_4_, 1 g NaCl, 1 g sodium citrate, and 0.5 g disodium citrate sesquihydrate. QuEChERS salt mixture for extract purification consists of 25 mg primary secondary amine (PSA), 45 mg graphitized carbon black (GCB), and 900 mg magnesium sulfate (MgSO_4_).

High-purity concentrated HNO_3_ (65% *w*/*w*, Scharlau, Turkey) and certified 30% H_2_O_2_ (Alkaloids, Skopje, North Macedonia) were used for metal analyses and sample digestion. Standard solutions for calibration were prepared by diluting a stock solution of 100 mg/L (Be, V, Co, Ni, Cu, As, Se, Sr, Mo, Cd, Sb, Ba, Pb, B, Al, Cr, Mn, Fe, Zn, Rb, Sn) from CPAchem.

##### Sample Preparation

The preparation of samples for the quantitative determination of pesticides is done by extracting the pesticides using the QuEChERS technique (Quick, Easy, Cheap, Effective, Rugged, Safe). Of the homogenized strawberry sample, 10 g was weighed into a polypropylene cuvette with a screw cap. Of the acetonitrile, 10 mL was added to the weighed sample and the cuvette was vortexed vigorously for 1 min. A mixture of QuEChERS salt (4 g MgSO4, 1 g NaCl, 1 g Na-citrate, 0.5 g disodium citrate sesquihydrate) was then added to the cuvette and the cuvette was again vortexed vigorously for 1 min to obtain a crude extract. The extracts obtained must be purified on GC-MS/MS and UPLC-MS/MS before analysis to reduce the concentration of co-extracts. 

For pesticide analysis by gas chromatography, the extract was purified by transferring 6 mL of the extract to a dSPE column containing a salt mixture of 25 mg primary secondary amine (PSA), 45 mg graphitized carbon black (GCB), and 900 mg magnesium sulfate (MgSO4).

Afterwards, 6 mL of the extract was transferred to a dSPE purification cuvette; the dSPE cuvette was vortexed vigorously for 1 min and then centrifuged at 3000 rpm for 5 min.

For pesticide analysis by liquid chromatography, 100 µL of the crude extract was diluted with 900 µL of ultra-pure water.

For metal analyses, all samples were homogenized and 0.5 g of a single sample was weighed. Then, 3 mL of HNO_3_ and 1 mL of H_2_O_2_ were added to each sample. UltraWAVE ECR from Milestone was used for microwave digestion. After digestion, the samples were diluted to 20 mL with deionized water.

##### GC-MS/MS and LC-MS/MS Analysis and ICP/MS Analysis

The pesticide content in the prepared sample was determined using gas and liquid chromatography, coupled with mass spectrometry, using the standard method (HRN EN 15662:2018) and monitoring selected reactions (MRM-multi-reaction monitoring). The gas chromatograph used was the Shimadzu GC-MS-TQ8050 NX coupled with mass spectrometry and the AOC 6000 autosampler. (Shimadzu, Japan). The analytes were chromatographically separated on a capillary column Rxi-5Sil MS manufactured by Restek (30 m × 0.25 mm inner diameter × 0.25 µm film thickness). Pesticide analysis by liquid chromatography was performed using the UPLC-MS/MS Waters Xevo TQ MS equipped with a thermostatted autosampler, column heater, MS degasser pump (Waters Xevo TQMS), nitrogen generator, and a computer data processing system. The analytes were chromatographically separated on an ACQUITY UPLC BEH C18 150 × 3.0 mm column; 1.7 μm (Waters corporation).

Identification and quantification were performed using a matrix-matched calibration in which a standard solution was prepared in a strawberry matrix (corresponding to the limit of quantification 0.01 mg kg^−1^). A strawberry sample that was found to contain pesticides below the limit of detection for recovery tests and for the matrix-matched standard was used. The homogenized blank sample was spiked to a concentration of 0.01 mg kg^−1^ by standard addition prior to the determination procedure. The recovery values must meet the critical limit of 80–120%. For analytes that have been shown to have satisfactory precision (RSD < 20%), a lower recovery may be accepted in accordance with SANTE/12682/2019. The matrix-match standard was analyzed for every 10 samples; the concentrations obtained must not differ by ±20%. 

Pesticides were identified based on retention time, a target ion, and two qualifier ions (tolerance ± 0.1 min for retention time). The ratio of the selected ion transitions in the sample must correspond to the ratio of the same ion transitions in the MM standards, with a tolerance of ± 30%. The maximum reporting limit (MRLs) of pesticides in strawberries were checked on the day the results were issued in Regulation 396/2005, https://ec.europa.eu/food/plant/pesticides/eu-pesticides-database/mrls/?event=search.pr (accessed on 25 October 2021). 

Inductively coupled plasma with mass spectrometry (Agilent 7900 ICP-MS), and high-purity argon and helium (≥99.99%) were used for metal analyses. The ICP-MS measurements were performed using the Micro Mist Nebulizer, with the Rf power, plasma, nebulizer, and auxiliary gas set to 1180 W, 15.0 L/min, 1.07 L/min, and 0.90 L min^−1^, respectively. Before the samples were measured, the instrument was calibrated. The reagent blank solution contained 1% HNO_3_. Mixed standard solutions were prepared in reagent blank solutions. Linearity was tested by injecting seven concentrations of the working standard. Each concentration was injected three times and the regression line and correlation coefficient were determined. A correlation coefficient of ≥0.99 was obtained for each element. The matrix effect was compensated for by adding internal standards (mixture 100 µg L^−1^ Bi, Ge, In, Li6, Sc, Tb, Y from Agilent) to the solutions.

The precision parameter was determined by preparing a sample (*n* = 6) multiple times before measuring each sample and giving it an RSD of 4.5%. The limits of quantification ranged from 0.01–0.25 mg kg^−1^.

#### 2.3.4. Sensory Evaluation 

Sensory analysis of the fresh strawberries (F1 and F2) and juices (J1 and J2) was performed using a Quantitative Descriptive Analysis (QDA), with a total of 13 sensory descriptors evaluated [19,33]. The descriptive terms are listed in Table 1. A team of 16 professional panelists from the Faculty of Agriculture and the Faculty of Food Technology and Biotechnology at the University of Zagreb, aged 20–52, was selected based on their sensory acuity, sensitivity, and ability to distinguish small differences in the intensity of a sensory attribute. Panelists scored the samples for each characteristic in the vocabulary, using an appropriate line intensity scale, with scores assigned on a scale of 0–7 to indicate the relative intensity of each attribute, with 0 indicating the complete absence (‘none’) of the sensory attribute and 7 indicating a very distinct attribute (‘intense’). Fresh fruit samples consisting of four fresh fruits with sepals removed were served on white porcelain plates, while juice samples (30 mL) were served in two identical cups. The panelists cleansed their mouths with salt-free bread and water between each sample.

#### 2.3.5. Determination of Bioactive Compounds (BACs)

All absorbance measurements were conducted with an UV/Vis spectrophotometer (LLG-uniSPEC 2 Spectrophotometer, Buch & Holm, Meckenheim, Germany). For each sample, duplicate measurements were performed. 

##### Extraction Procedure

The extraction of bioactive compounds from fresh strawberry fruits (F1 and F2), strawberry juices (J1 and J2), and strawberry by-products (BP1 and BP2) was performed according to modified protocols from the literature [34]. Briefly, 5 g of the sample were mixed with 20 mL of 1% formic acid in 80% methanol (*v*/*v*). The mixture was vortexed for 1 min and extracted for 15 min at 50 °C in an ultrasonic bath (DT 514 H SONOREX DIGITEC 13.5 L, 860 W, 40 kHz, Bandelin electronic, Germany). The mixture was then centrifuged at 10,000 rpm/10 min (Thermo Scientific™, Megafuge™ 16R, Kalkberg, Germany) and the supernatant was filtered through Whatman filter paper No. 40 (Whatman International Ltd., Kent, UK), and increased to 25 mL in volumetric flask using an extraction solvent. All extracts were prepared in duplicate. Prior to analysis, the extracts were stored at −18 °C in an inert gas atmosphere.

##### Determination of Total Phenolic Content (TPC) 

Total phenolic content was determined using a modified spectrophotometric method described in the literature [35]. Briefly, 400 µL of the properly diluted extract was mixed with 400 µL of FC reagent (previously diluted 5 times with distilled water) and 4 mL of 7.5% sodium carbonate solution (*w*/*v*). The reaction mixture was allowed to stand at room temperature for 20 min and the absorbance was measured at 725 nm using a spectrophotometer. A calibration curve was prepared using a standard solution of gallic acid (10–250 mg L^−1^) and the results were expressed as mg gallic acid equivalent (GAE) per 100 g or 100 mL of the sample.

##### Determination of Total Hydroxycinnamic Acids (HCA) and Total Flavonols (TF)

HCA and FL were determined using a modified spectrophotometric assay [36]. Briefly, 250 μL of extract and 250 μL of solution were stirred in a vortex for 10 s and then allowed to react in the dark at room temperature for 30 min. The absorbance of the solution was then measured at 320 nm for HCA and 360 nm for FL in a spectrophotometer. A blank was prepared in the same way, but an extraction solvent was used instead of the extract.

For quantification of HCA, a standard solution of chlorogenic acid (10–600 mg L^−1^) was used to prepare the calibration curve and the results were expressed as mg chlorogenic acid equivalent (CAE) per 100 g or 100 mL of the sample. For the quantification of FL, a standard solution of quercetin (10–600 mg L^−1^) was used to construct the calibration curve and the results were expressed as mg quercetin equivalent (QE) per 100 g or 100 mL of the sample.

##### Determination of Monomeric Anthocyanins (MA)

The determination of MA was performed using a spectrophotometric pH differential method [37]. Briefly, 1 mL of the extract was mixed with 4 mL of 0.025 M potassium chloride buffer (pH 1.0) and also separately with 4 mL of 0.4 M sodium acetate buffer (pH 4.5). The reaction mixture was allowed to stand at room temperature for 20 min and absorbance was measured using a spectrophotometer at 520 nm and 700 nm, using deionized water as a blank. The concentration of monomeric anthocyanins in the sample was calculated as equivalent of pelargonidin-3-glucoside (Pg-3-G) (mg L^−1^) according to Formula (6):

(6) $$\;\frac{A\;\times \mathrm{MW}\;\times \mathrm{DF}\;\times 10^{3}}{\varepsilon \times l}$$

where: A = (A_520nm_ − A_700nm_)_pH=1.0_ − (A_520nm_ − A_700nm_)_pH=4.5_; MW = molecular weight (for pelargonidine-3-glucoside C_21_H_21_ClO_10_ = 468.8 g mol^−1^); DF = dilution factor; 10^3^ = factor for conversion g to mg; ε = molar absorption extinction coefficient (for pelargonidine-3-glucoside 22,400 L mol^−1^ cm^−1^); l = cuvette thickness (1 cm).

#### 2.3.6. Statistical Analysis

Descriptive statistics were used for the characterization of the sample. Discrete variables and factor scores were tested using MANOVA. Ward’s method of exploratory hierarchical cluster analysis was used for measuring standardized similarities in the samples. The level of significance for all tests was α ≤ 0.05, and results were analyzed using SPSS software (v.22).

## 3. Results and Discussion

### 3.1. Physiochemical Assessment of Strawberry Fruits

The results for changes in the physiochemical parameters in strawberry cv. ‘Albion’ at different levels of maturity and storage are shown in Table 2. As can be seen, the average mass of sampled strawberries was 51.79 ± 1.07 g. As expected, the fruit weight with the calyx was almost 40% higher in strawberries at full maturity, while this mass decreased by 5% from the baseline at 4 days of storage. The different degree of ripeness did not affect this parameter during storage. The average calyx mass was 0.80 ± 0.03 g. Here, the weight of the calyces of the fully ripe strawberries was 37% higher than that for fruits with a lower degree of ripeness, while their mass decreased by 25% during storage. The differences in the ripeness of the fruits did not affect the loss of calyx mass during storage. Next, the mass of strawberry fruits without calyces was 23% higher in the fully ripe samples than in their less ripe counterparts, while it remained constant during 4 days of storage. Their average mass in the data set was 50.99 ± 1.05 g.

The maximum acceptable weigh loss for strawberries stored at 20 °C and 85–95% relative humidity was 2.5–3% within 2.5–3 days, resulting in a softening of the flesh, darkening of the color, and drying of the calyx and skin [17]. In a study by Kelly et al. [38], weight loss of strawberries during 9 days of storage at 1.5 °C was also found to be in the range of 2.97–5.97%. The reduction in fruit weight during storage is caused by the loss of moisture from the fruit, which may have a negative effect on their appearance and processing costs. The morphological characteristics of the fruit, its size, and initial moisture content, as well as the integrity of the skin, affect the rate of moisture loss from the fruit [38]. 

The average firmness of the samples was 0.40 ± 0.14 kg cm^−2^. Samples that were less ripe had 35% higher firmness than fully ripe samples, while total hardness increased by 20% during storage. Moreover, there was a significant decrease in hardness during storage of strawberries, making them softer and more susceptible to spoilage, which is in agreement with other literature data [22,39,40].

The average TSS in the samples was 8.98 ± 0.14° Brix, which is slightly higher than the values obtained by Ornelas-Paz [39] for the same cultivar, where higher TSS content was found in strawberries at a higher maturity stage compared to fruits with lower maturity. About a 5% higher level of soluble solids content was found in fully ripe fruits, while it decreased by nearly 7% during storage. Other researchers also confirmed that TSS increases with fruit ripeness [22,39,40]. Similarly, TSS in strawberry fruit decreases during storage of 9 days at 1.5 °C. However, the relationship between the ripeness levels of strawberries and the days of storage was not statistically confirmed [38].

Fully ripe strawberries had a slightly higher pH, which tended to decrease during storage. This parameter averaged 3.25 ± 0.01 in the samples. Other researchers also confirmed that pH increased with fruit ripeness [22,39]. Olsson et al. noted a decrease in pH in ripe strawberries after 3 days of storage at +4 °C. A possible explanation for this trend could be that the low temperature contributes to the stabilization of pH during storage [41]. On the other hand, the total acidity was about 21% lower in more mature samples, which essentially means that the total acidity decreased by 1% for almost every percent of ripeness. In contrast to SSC, titratable acidity tended to decrease during maturation [22,39]. Storage had a significant effect on total acidity, which tended to decrease by 12% during 4 days of storage, which is in agreement with the results of Kelly et al. [38]. As for the other parameters, their pH and TA were not altered by the simultaneous influence of ripening and storage. 

In conclusion, the obtained results show that the storage of fresh strawberries intended for processing is significantly affected by the degree of ripeness and storage time; therefore, these parameters must be carefully considered in order to produce a functional strawberry juice of the highest quality.

### 3.2. Colorimetric Assessment of Strawberry Fruits

The color of fresh strawberries, as well as strawberry products, is one of the most important quality indicators that consumers percieve first [25]. Therefore, in this work, the color parameters of fresh strawberries were monitored on the day of harvest and during a 4-day storage at 4 °C (Table 3). On average, all samples were in the darker, more reddish–yellow range of the CIELAB light spectrum. At the same time, the samples with a lower degree of ripeness were 14% brighter than their fully ripe counterparts. They also had a 13% higher a* value and an 18% higher b* value, while chroma was 17% higher in these samples. There was no difference in hue between the fully ripe and 75% ripe fruits.

During storage, the fruits became darker, while other CIELAB parameters remained the same. Changes in strawberry fruit color are most commonly observed during fruit ripening, while the results of studies comparing strawberry fruit color before and after storage for different ripening stages have not been found. However, a possible explanation for this observed trend could be the decomposition of hexoses during storage, which is more pronounced in fruits with higher ripeness (higher SSC) due to the Maillard reaction, which may consequently manifest itself in the darkening of the color of the strawberry [42].

Interestingly, the color changes seem to be more pronounced in the already fully ripe fruits because in 75% of the ripe strawberries, L*, a*, and H* did not change during storage, while a change was only observed in parameter b*, shifting the less ripe fruits towards the yellowish part of the spectrum. On the other hand, the fully ripe fruits showed changes in all CIELAB variables except a*. Here, the fully ripe fruits became 13% darker and moved away from the yellowish part of the spectrum (21%), while hue and chroma decreased by 12% and 14%, respectively.

Finally, it is interesting to note that the color change (ΔE) is greater in fully ripe strawberries (6.07) than in strawberries with a lower degree of ripeness (3.85), implying that less ripe strawberries, if they meet other quality parameters, would potentially be more interesting for processing [22,40]. 

### 3.3. Physiochemical and Color Assessment of Strawberry Juices

Stored strawberries of both ripening stages were processed into juice and the yield and the physicochemical analysis of all samples were determined. Because the goal was to produce functional strawberry juices, cold pressing was chosen as the technology for juice production, as this technology produces thick and pulpy juices with no temperature rise during the production process. Considering the yield of the process in terms of juice production, the results showed that strawberries of both ripening stages had a similar yield (68.47% at 75% ripeness vs. 69.87% at 100% ripeness) (Table 4). These results confirm that strawberries from both maturity levels are suitable for processing.

Previous studies have confirmed that the yield of strawberries processed into juice is highly dependent on the cultivar, with the authors obtaining juice yields ranging from 48.22% to 89.98% for 15 different strawberry cultivars [43]. 

Without the influence of ripeness, the average SSC and pH of the juice samples were 8.38 ± 0.06 and 3.31 ± 0.01, respectively. Juices from less ripe fruits (75%) had TSS and pH levels of 7.85 ± 0.08 and 3.22 ± 0.02, respectively, while for fully ripe fruits, both parameters were significantly higher and were 8.90 ± 0.08 and 3.39 ± 0.02, respectively. However, comparing these values for juice with the results for fresh fruits of the same degree of ripeness, it is observed that during processing into juice, both the TSS and pH of the juice decreased.

### 3.4. Toxicology Analysis 

#### 3.4.1. Heavy Metals

Analysis of heavy metals (Cu, Zn, Ni, As, Cd, Pb) in fresh strawberry fruit, juice, and by-product samples showed that the concentrations for Ni and Cd were below the detection limits. The ICP-MS method (Table 5) detected the following metals Cu (0.077–0.415 mg kg^−1^), Zn (0.988–3.12 mg kg^−1^), As (<0.02–0.04 mg kg^−1^) and Pb (<0.03–0.076 mg kg^−1^), which is in agreement with the results of a group of authors from China who found lead, cadmium, and nickel in most strawberry samples, with detection rates of 75.76, 92.93, and 92.93%, respectively [44]. In a paper from Poland, a group of authors found that the average content of heavy metals in strawberry fruits grown in the Lublin region was 0.023 mg Pb, 0.020 mg Cd, 0.091 mg Ni, 1.228 mg Zn, 0.358 mg Cu, 0.0015 mg As, and 0.00011 mg Hg per kg of fresh weight indicating that the threshold for products of this type was not exceeded [45].

Among 250 samples of fruit and vegetable products from the Libyan market, the highest Pb concentrations were found in mangoes, followed by strawberries (0.53 ± 0.2 mg kg^−1^). Moreover, the authors detected several heavy metals in the strawberry samples: Cd 0.01 ± 0.02 mg kg^−1^, Ni 1.818 ± 0.103 mg kg^−1^, Zn 1.32 ± 3.12 mg kg^−1^, Cu 3.14 ± 0.58 mg kg^−1^, and Co 0.272 ± 0.58 mg kg^−1^ [46].

In 2021, new, stricter MRLs for Pb and Cd in food came into force in the EU, amending Commission Regulation (EC) No 1881/2006 of 19 December 2006, setting maximum levels for certain contaminants in foodstuffs [47], with Commission Regulation (EU) 2021/1317 of 9 August 2021 amending Regulation (EC) No 1881/2006 [48] regarding maximum levels for lead in certain foodstuffs, and Commission Regulation (EU) 2021/1323 of 10 August 2021 amending Regulation (EC) No 1881/2006 regarding maximum levels for cadmium in certain foodstuffs. [49].

In samples of fresh strawberries fruits, Pb was not found, but in sample J2 strawberry juice from fruits of 100% ripeness, the level for Pb exceeded the permissible MDK for juice. The concentration in sample BP2 from fruits of 100% ripeness was even higher than in J2, but below the MDK for strawberries fruits. On 18 March 2010, the European Food Safety Authority (‘the Authority’) adopted an opinion on lead in food [50]. The Authority found that lead may cause developmental neurotoxicity in young children and cardiovascular problems and nephrotoxicity in adults. The risk assessment for lead was based on these potentially critical adverse effects.

#### 3.4.2. Pesticides

The strawberry is a perishable fruit that is easily attacked by fungi after harvest, so it is often treated with fungicides, cyprodinil, pyrimethanil, and fludioxonil being the most commonly used [51,52,53]. Pyrimethanil and cyprodinil belong to the class of anilinopyrimidine fungicides that prevent protein formation and cell division in fungal pathogens (such as gray mold, powdery mildew, scab, downy mildew, and Phomopsis leaf spot on a variety of crops, including apples, oranges, strawberries, root crops, and tubers) by inhibiting methionine biosynthesis [54,55]. Botrytis fruit rot, caused by *Botrytis cinerea*, is one of the most threatening strawberry diseases worldwide. To control the disease, fungicides containing pyrimethanil or cyprodinil as active ingredients are commonly used in commercial strawberry production [55]. Pyrimethanil and cyprodinil have low acute toxicity to humans, but there are some toxicological concerns related to their antiandrogenic properties [54,55,56,57,58,59,60]. According to the PubChem open chemistry database, pyrimethanil is classified as a Group C ‘possible human carcinogen,’ while there is no evidence of carcinogenic potential for cyprodinil at any dose [45]. Both substances are toxic to aquatic organisms [53], and they are both toxic to aquatic life [53,61]. The results from the literature indicate that the risk of using pyrimethanil in strawberries at the recommended dosage is negligible for humans [60]. 

The strawberry samples were analyzed for 261 pesticides at GC-MS/MS and 305 pesticides at LC-MS/MS. The pesticides detected in the strawberry samples were cyprodinil and pyrimethanil (LC-MS/MS). No pesticides were detected using GC-MS/MS (Table 6). The results are listed with the measurement uncertainty for each result.

The MRL for cyprodinil and pyrimethanil in strawberries given in the EU database is 5 mg kg^−1^. Strawberries are included in the ‘high acidity and water content’ category [62]. Cyprodinil was detected only in the by-product BP1by LC-MS/MS. The average recovery for cyprodinil in strawberries is 68% and the RSD for sample preparation repeatability is 4.2%. Since the preparation repeatability < is 20%, this recovery can be accepted, but the results for cyprodinil in strawberries need to be corrected based on the recovery. The average recovery for pyrimethanil in the ‘high acidity and water content’ commodity group, which includes strawberries, is 109%. This means that the results do not need to be corrected for recovery. Overall, it can be concluded that the processed raw materials and juices were toxicologically safe.

### 3.5. Sensorial Comparison of Strawberry Fruits and Juices

The sensory characteristics of fresh fruit can be degraded when processed into juice, affecting consumers’ sensory perception of the final products [63,64]. In this study, the fresh samples and corresponding juices were sensory evaluated using 13 sensory descriptors and the results are shown in Table 7.

The average color intensity score of the samples was 5.66/7, representing 81% of the total score. The testers were not able to identify the difference in color intensity of the juices compared to the fresh fruits, as they gave them the same score with an average of 5.70. However, they were able to distinguish different ripeness levels as they gave 38% fewer points for less ripe samples and opposed to the riper samples (4.75 vs. 6.56). On the other hand, panelists were unable to differentiate the redness of the fruit processed into juice by ripeness. Next, the taste of all samples (fruits and juices) was assessed with 72% of the total points. Similar to color, panelists gave 13% more points to samples with lower ripeness. However, as before, they were unable to consistently distinguish the flavor intensity of the fruits and juices with different levels of ripeness. On average, fruit flavor received 77% of the total score, while panelists were unable to consistently distinguish the degree of floral flavor among fruits and juices. However, they were able to identify a 22% higher intensity of fruit flavor in fully ripe samples than in samples with a ripeness score of only 75%. The average intensity of greenish flavor in the samples was 41% of the total score. For the fruits and juices, this intensity was the same, averaging 2.88 points. The testers evaluated the intensity of greenish flavor by 56% in samples with lower ripeness, but were not able to evaluate the intensity of this flavor in relation to ripeness and conversion in strawberry juices. The intensity of off-flavor and beige flavor in the samples was 19% and 20% of the maximum value, respectively, although this was not related to the type of samples, i.e., whether they were fruits or juices, and independent of the degree of ripeness and their mutual influences. The greater presence of more intense sour or green taste attributes may be attributed to a higher concentration of acid in unripe fruits, along with a lower presence of sugars [39]. Therefore, in determining the perceived sweet and sour taste of strawberries, the ratio of sugar to acid plays an important role [39].

The average value for flavor intensity and sour taste of the samples was 75% and 57% of the maximum intensity, respectively. Ripeness was the only thing panelists could relate to the difference in flavor intensity and sourness, so they gave 18% fewer points to samples with lower ripeness. Interestingly, samples with full ripeness were rated as more acidic than their 75% ripe counterparts. However, neither flavor intensity nor acidity were related to the mutual influence of ripeness and whether or not the samples were fruits or juices. Ripeness was another parameter by which panelists could distinguish the intensity of sweet and harmonic tasting samples, but as with other samples, the ability to associate different levels of ripeness with fruits and juices was not evident. Here, lower ripeness samples had 27% lower intensity for sweetness and 24% for harmony than the fully ripe samples. Overall, the intensity for these two parameters was 64% and 70% of the total intensity, respectively. Thus, an increase in sugar content and a simultaneous decrease in organic acids should lead to an increased perception of sweetness in ripe strawberries [39,65].

In addition, texture (as firmness for fresh samples and as homogeneity for juice samples) was rated as 79% of the total score for all samples, and this rating was similar for all samples of fruits and juices, regardless of ripeness. As expected, ripeness was again the only variable that allowed testers to distinguish between samples, as they gave fully ripened samples a 20% higher score for texture, while this score did not vary for other fruits and juices, either alone or in combination with ripeness.

Finally, the overall sensory quality of all samples was rated as 75% of the total score. As with the rest of the dataset, ripeness was the only parameter associated with overall sensory quality. Here, more mature samples received almost 20% more points than the less mature samples. This parameter did not differ significantly between juices and fruits with different degrees of ripeness. In other words, juices made of 75% and 100% ripe fruits were practically indistinguishable by the consumers.

### 3.6. Biologically Active Compounds in Fresh Strawberries, Their Juices, and By-Products

The highest content of all bioactive compounds (BAC) was found in strawberry by-products and the lowest in fruit juices (Table 8). This is a very interesting result, as it indicates the great importance of the use of strawberry by-products in food production. By-products contained either the same or higher amounts of all the tested BACs when compared to raw fruits or juices. This was particularly evident in total flavonols (FL), which were 11 times higher in by-product samples than in juices. Total polyphenols (TPC) and especially anthocyanins (ANT) contents did not differ from that in raw fruits. 

Higher ripeness was associated with lower levels of TPC, higher levels of ANT, lower levels of hydroxycinnamic acids (HCA), and no difference in FL. This relationship was maintained only for HCA in the fruit samples tested, while the other BACs remained the same in the other raw fruit samples. ANT maintained this relationship when raw strawberries were converted to juices, but with a greater discrepancy in the concentration between the lower and fully ripened samples than previously noted in the raw fruit samples. This may be supported by the fact that the phenolic components are mainly synthesized in the skin of the fruit; therefore, their content decreases with increasing fruit weight or ripeness [66]. Thus, the content of phenolic compounds is higher in some unripe fruits, such as grapes [67], kiwifruit [68], apples [69], and pomegranates [70], than in ripe fruits. Juice production requires the disruption of fruit cells to release the juice, which is usually achieved by mechanical action on the fruit [57]. Since the hardness of the fruit decreases as it ripens [22,39,40,71], it is possible to achieve better cell disruption and ultimately, better extraction, of water-soluble components such as anthocyanins. This would be a possible explanation for the results obtained.

Interestingly, the increase/decrease in HCA content and ripeness reversed after conversion to juices. Here, the HCA content increased in the juices prepared from fruits with full ripeness, while this relationship was completely lost in the by-products. This means that all samples of the by-products had the same HCA content, regardless of ripeness. As with anthocyanins, a possible explanation could be the lower tissue hardness of fully ripe fruits compared to 75% ripe fruits and the consequent better extraction of HCA components [71].

The processing of the fruit into juice was the only factor that affected the content of FL in the samples, while the ripeness of the fruit had no effect on FL. This was also true when the dataset was broken down by the type of material assessed (e.g., fruits, juices, or by-products).

**Figure 3:**
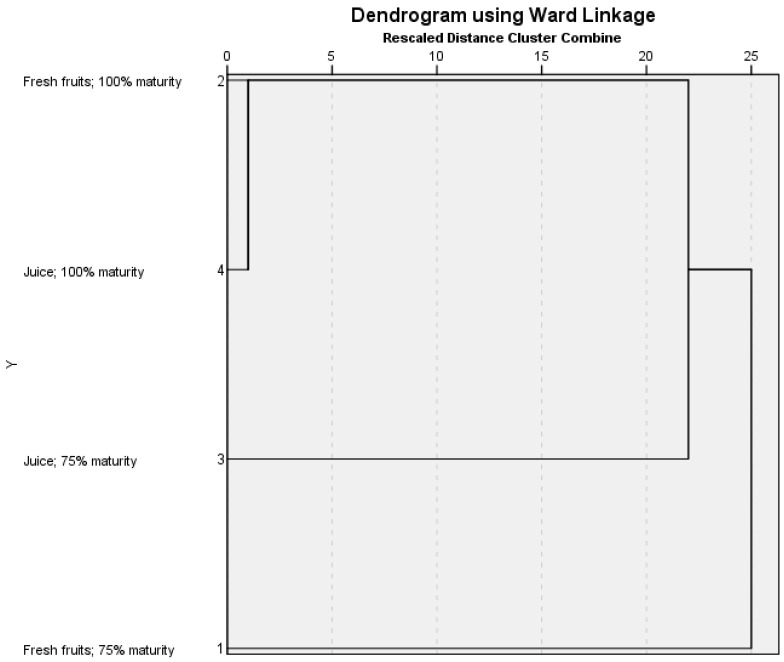
Clustering of samples of strawberry fruits and juices with various maturity with regards to average values for CIELAB L*; a*; b*; C*; H*; pH; contents of total phenolic compounds (mg GAE 100 mL^−1^); anthocyanins (mg Pg-3–100 mL^−1^); hydroxycinnamic acids (mg CAE 100 mL^−1^); flavonols (mg QE 100 mL^−1^); and overall sensory quality.

A hierarchical cluster analysis using the standardized Ward’s method revealed that when considering average values for CIELAB L*; a*; b*; C*; H*; pH; contents of total phenolic compounds (mg GAE 100 mL^−1^); anthocyanins (mg Pg-3–100 mL^−1^); hydroxycinnamic acids (mg CAE 100 mL^−1^); flavonols (mg QE 100 mL^−1^); and overall sensory quality at different ripeness levels of the fruits and their juices, the results were clustered in an interesting manner. As expected, the samples of the juices and the corresponding fruits at 100% ripeness were clustered closely together. However, the next-closest neighbors included samples of juices at 75% maturity, suggesting that even at lower maturity, strawberry juices were similar to those of 100% maturity (see Figure 3). 

Further away from this cluster were fruits with a ripeness level of 75%. Although still close to the corresponding juice samples with 75% ripeness, they are outside the cluster that includes fruits and juices with 100% ripeness. In other words, it appears that the strawberries with lower ripeness, when processed into juices, are of similar quality to fruit with higher ripeness. This could be important for industrial growers and juice processors, saving them the time it takes strawberries to reach full maturity from the 75% mark on, allowing them to harvest earlier without any particular loss of quality. Moreover, in comparison to 75% ripe fruits, the lower mechanical strength and susceptibility to fungal attack of fully ripe strawberries can be a limiting factor in production, especially in the transportation and marketing stages, resulting in significant losses [72,73]. The perspective of the use of unripe fruits is also being increasingly considered for various industrial purposes, for example, thinning of unripe fruits has shown great potential in food processing, bringing economic benefits and reducing environmental impact [66].

## 4. Conclusions

It has been found that the degree of fruit ripeness has a greater influence on the color characteristics of fresh fruit than does storage, implying that less ripe ‘Albion’ strawberries, if they meet other quality parameters, could be suitable for processing.

Out of the analyzed concentrations of detected heavy metals (Cu, Zn, Ni, As, Cd, Pb) in strawberries, the permissible values assigned (MDK) to these types of products were not exceeded, but in the juice and by-product, there was a slightly higher amount of Pb with 100% ripeness, while the other metal concentrations were consistent with the results in the literature. Cd and Ni were not detected in any sample. Of the 566 pesticides analyzed, only two pesticides were detected with LC-MS/MS, cyprodinil and pyrimethanil.

As for the sensory analysis, the fresh fruits did not differ from the corresponding juices in any of the sensory attributes studied, confirming that sensory perception was not affected during the processing of the fresh fruits into juice. Nevertheless, ripeness proved to be a significant factor influencing most sensory attributes, with the 100% ripe fruits and their juices exhibiting an almost 20% higher overall sensory quality than the less ripe fruits and their juices.

The highest content of all bioactive compounds (BAC) was found in strawberry by-products and the lowest in fruit juices, indicating the great importance of using by-products in the production of functional foods.

A chemometric evaluation was successfully applied to strawberry cv. ‘Albion’ for processing into functional juice in terms of physicochemical parameters, sensory analysis, and bioactive compounds. The results confirmed that, in addition to fully ripe fruits, strawberries with a lower degree of ripeness (75%) are also suitable for processing with respect to all quality parameters evaluated. These results are important for industrial juice producers because strawberries are a very delicate fruit with a tender texture that is easily damaged during transportation and storage. Therefore, by processing less ripe strawberries that better survive transport and storage due to their better textural properties, high-quality functional strawberry juices could also be produced.

## Figures and Tables

**Table 1:** Sensory attributes used for sensory evaluation.

Sensory Attribute	Descriptive Term
Color	Color intensity
Flavor	Flavor intensity
	Floral flavor
	Fruity flavor
	Green flavor
	Off-flavor
Taste	Taste intensity
	Acid taste
	Sweet taste
	Harmonious
	Off-taste
Texture	Firmness/homogeneity
Overall sensory quality	Overall sensory quality

**Table 2:** The changes of the physiochemical parameters in strawberry cv. ‘Albion’ with different ripeness levels and storage.

Strawberry Fruit Parameters	M1	M2	M3	H_avg_	TSS	pH	TA
Maturity	*p* ≤ 0.01 ^†^	*p* ≤ 0.01 ^†^	*p* ≤ 0.01 ^†^	*p* ≤ 0.01 ^†^	*p* = 0.09 ^‡^	*p* ≤ 0.01 ^†^	*p* ≤ 0.01 ^†^
F1	43.34 ± 1.52 ^b^	0.68 ± 0.04 ^b^	42.67 ± 1.49 ^b^	0.48 ± 0.02 ^a^	8.75 ± 0.19 ^a^	3.25 ± 0.01 ^b^	1.10 ± 0.01 ^a^
F2	60.23 ± 1.52 ^a^	0.93 ± 0.04 ^a^	59.30 ± 1.49 ^a^	0.31 ± 0.02 ^b^	9.22 ± 0.19 ^a^	3.38 ± 0.01 ^a^	0.87 ± 0.01 ^b^
Storage	*p* = 0.24 ^‡^	*p* ≤ 0.01 ^†^	*p* = 0.27 ^‡^	*p* = 0.02 ^†^	*p* = 0.02 ^†^	*p* ≤ 0.01 ^†^	*p* ≤ 0.01 ^†^
0 days	53.08 ± 1.52 ^a^	0.92 ± 0.04 ^a^	52.17 ± 1.49 ^a^	0.43 ± 0.02 ^a^	9.32 ± 0.19 ^a^	3.38 ± 0.01 ^a^	1.05 ± 0.01 ^a^
4 days	50.50 ± 1.52 ^a^	0.69 ± 0.04 ^b^	49.80 ± 1.49 ^a^	0.36 ± 0.02 ^b^	8.65 ± 0.19 ^b^	3.26 ± 0.01 ^b^	0.92 ± 0.01 ^b^
Maturity by Storage	*p* = 0.79 ^‡^	*p* = 0.67 ^‡^	*p* = 0.79 ^‡^	*p* = 0.17 ^‡^	*p* = 0.34 ^‡^	*p* = 0.10 ^‡^	*p* = 0.70 ^‡^
F1; 0 days	44.35 ± 2.14 ^a^	0.78 ± 0.06 ^a^	43.57 ± 2.1 ^a^	0.43 ± 0.03 ^a^	8.95 ± 0.27 ^a^	3.30 ± 0.01 ^a^	1.16 ± 0.01 ^a^
F1; 4 days	42.34 ± 2.14 ^a^	0.58 ± 0.06 ^a^	41.77 ± 2.1 ^a^	0.54 ± 0.03 ^a^	8.54 ± 0.27 ^a^	3.21 ± 0.01 ^a^	1.04 ± 0.01 ^a^
Maturity by Storage	*p* = 0.79 ^‡^	*p* = 0.67 ^‡^	*p* = 0.79 ^‡^	*p* = 0.17 ^‡^	*p* = 0.34 ^‡^	*p* = 0.10 ^‡^	*p* = 0.70 ^‡^
F2; 0 days	61.82 ± 2.14 ^a^	1.06 ± 0.06 ^a^	60.76 ± 2.1 ^a^	0.30 ± 0.03 ^a^	9.69 ± 0.27 ^a^	3.45 ± 0.01 ^a^	0.93 ± 0.01 ^a^
F2; 4 days	58.65 ± 2.14 ^a^	0.81 ± 0.06 ^a^	57.84 ± 2.1 ^a^	0.32 ± 0.03 ^a^	8.75 ± 0.27 ^a^	3.31 ± 0.01 ^a^	0.81 ± 0.01 ^a^
Average in samples	51.79 ± 1.07	0.80 ± 0.03	50.99 ± 1.05	0.40 ± 0.14	8.98 ± 0.14	3.25 ± 0.01	0.98 ± 0.01

F1—fresh fruits with 75% maturity; F2—fresh fruits with 100% maturity. Results are expressed as mean ± standard error. Values represented with different letters are statistically different at *p* ≤ 0.05; ^†^—significant factor in multifactor analysis; ^‡^—not significant factor in multifactor analysis. M1—Fruit weight with calyx (g); M2—Calyx weight (g); M3—Fruit weight without calyx (g); H_avg_—Average value for hardness measured from frontal and rear part of the fruit (kg/cm^−2^); TSS—Total Soluble Solids (% Brix). TA—total acidity (%); For M1–M4; H_avg_ and SSC *n* = 40 for pH, and TA *n* = 12.

**Table 3:** Changes in the CIELAB parameters in strawberry cv. ‘Albion’ at different stages of ripeness and storage.

Strawberry Fruit Parameters	*n*	L*	a*	b*	C*	H*
Maturity		*p* ≤ 0.01 ^†^	*p* ≤ 0.01 ^†^	*p* ≤ 0.01 ^†^	*p* ≤ 0.01 ^†^	*p* = 0.55 ^‡^
F1	40	33.83 ± 0.49 ^a^	19.15 ± 0.39 ^a^	22.98 ± 0.83 ^a^	30.15 ± 0.67 ^a^	49.43 ± 1.35 ^a^
F2	40	29.68 ± 0.49 ^b^	16.55 ± 0.39 ^b^	19.33 ± 0.83 ^b^	25.79 ± 0.67 ^b^	48.29 ± 1.35 ^a^
Storage		*p* ≤ 0.01 ^†^	*p* = 0.91 ^‡^	*p* = 0.72 ^‡^	*p* = 0.81 ^‡^	*p* = 0.42 ^‡^
0 days	40	33.18 ± 0.49 ^a^	17.82 ± 0.39 ^a^	21.36 ± 0.83 ^a^	28.09 ± 0.67 ^a^	49.64 ± 1.35 ^a^
4 days	40	30.33 ± 0.49 ^b^	17.88 ± 0.39 ^a^	20.94 ± 0.83 ^a^	27.86 ± 0.67 ^a^	48.08 ± 1.35 ^a^
Maturity by Storage		*p* = 0.17 ^‡^	*p* = 0.89 ^‡^	*p* = 0.04^†^	*p* = 0.05^†^	*p* = 0.64 ^‡^
F1; 0 days	20	34.51 ± 0.69 ^a^	19.08 ± 0.55 ^a^	21.18 ± 1.17 ^a^	28.75 ± 0.95 ^b^	47.23 ± 1.91 ^a^
F1; 4 days	20	33.15 ± 0.69 ^a^	19.22 ± 0.55 ^a^	24.78 ± 1.17 ^b^	31.55 ± 0.95 ^a^	51.63 ± 1.91 ^a^
Maturity by Storage		*p* ≤ 0.01 ^†^	*p* = 0.89 ^‡^	*p* ≤ 0.01 ^†^	*p* = 0.02 ^†^	*p* = 0.02 ^†^
F2; 0 days	20	31.84 ± 0.70 ^a^	16.56 ± 0.55 ^a^	21.55 ± 1.17 ^a^	27.43 ± 0.95 ^a^	52.05 ± 1.91 ^a^
F2; 4 days	20	27.51 ± 0.70 ^b^	16.54 ± 0.55 ^a^	17.10 ± 1.17 ^b^	24.16 ± 0.95 ^b^	44.53 ± 1.91 ^b^
Average	80	31.75 ± 0.35	17.85 ± 0.27	21.15 ± 0.58	27.97 ± 0.48	48.86 ± 0.96

Results are expressed as mean ± standard error. Values represented with different letters are statistically different at *p* ≤ 0.05; ^†^—significant factor in multifactor analysis; ^‡^—not significant factor in multifactor analysis. L*—CIELAB lightness; a*—CIELAB green–red parameter; b*—CIELAB blue–yellow parameter; C*—CIELAB chroma; H*—CIELAB hue.

**Table 4:** Yield in the production of strawberry juice.

Parameter	Ripeness 75%	Ripeness 100%
Strawberry mass with calyces (g)	1004.73	1011.72
Strawberry mass without calyces (g)	989.73	998.37
Calyx weight (g)	14.74	13.12
Juice weight (g)	677.68	697.61
Pomace weight (g)	304.04	316.03
Proportion of calyces in relation to whole strawberries (%)	1.47	1.30
Proportion of juice in relation to strawberries with calyces (%)	67.45	68.95
Proportion of stalks in relation to strawberries without calyces (%)	68.47	69.87
Proportion of pomace in relation to strawberries with calyces (%)	30.26	31.24
Proportion of pomace in relation to strawberries without calyces (%)	30.72	31.66

**Table 5:** The concentration of heavy metals in the fresh strawberry, strawberry juice, and by-product samples.

Sample	Cu *m*/*z* 63	Zn *m*/*z* 67	Ni *m*/*z* 60	As *m*/*z* 75	Cd *m*/*z* 111	Pb *m*/*z* 208
F1	0.159 ± 0.006	1.20 ± 0.015	<0.04 ± 0.000	0.022 ± 0.009	<0.01 ± 0.000	<0.03 ± 0.000
F2	0.144 ± 0.008	1.24 ± 0.026	<0.04 ± 0.000	0.026 ± 0.008	<0.01 ± 0.000	<0.03 ± 0.000
J1	0.077 ± 0.003	1.03 ± 0.049	<0.04 ± 0.000	<0.02 ± 0.000	<0.01 ± 0.000	<0.03 ± 0.000
J2	0.132 ± 0.000	0.988 ± 0.033	<0.04 ± 0.000	0.038 ± 0.007	<0.01 ± 0.000	0.035 ± 0.002
BP1	0.412 ± 0.011	3.12 ± 0.091	<0.04 ± 0.000	0.040 ± 0.007	<0.01 ± 0.000	<0.03 ± 0.000
BP2	0.371 ± 0.003	2.40 ± 0.018	<0.04 ± 0.000	<0.02 ± 0.000	<0.01 ± 0.000	0.076 ± 0.005

F—fresh strawberry fruit; J—strawberry juice; BP—strawberry by-product; 1—ripeness 75%; 2—ripeness 100%. Results are expressed as the mean concentration ± standard deviation in mg kg^−1^ (*n* = 3).

**Table 6:** Pesticides detected in the strawberry samples (mg kg^−1^).

Pesticides/RT (min)/Recovery%	F1	F2	J1	J2	BP1	BP2
Cyprodinil/ 13.77/68	ND	ND	ND	ND	0.013 ± 0.0065	ND
Pyrimethanil/ 10.06/109	0.037 ± 0.0185	0.035 ± 0.0175	0.033 ± 0.165	0.034 ± 0.017	0.060 ± 0.030	0.053 ± 0.0265

F—fresh strawberry fruit; J—strawberry juice; BP—strawberry by-product; 1—ripeness 75%; 2—ripeness 100%. Results are expressed as the mean concentration ± measurement uncertainty in mg kg^−1^ (*n* = 6). ND—not determined.

**Table 7:** Results for the sensorial comparison of strawberry fresh fruits and corresponding juices.

Strawberry Fruit Parameters	*n*	S1	S2	S3	S4	S5	S6	S7	S8	S9	S10	S11	S12	S13
Material		*p* = 0.55 ^‡^	*p* = 0.85 ^‡^	*p* = 0.32 ^‡^	*p* = 0.85 ^‡^	*p* = 0.82 ^‡^	*p* = 0.40 ^‡^	*p* = 0.06 ^‡^	*p* = 0.75 ^‡^	*p* = 0.45 ^‡^	*p* = 0.11 ^‡^	*p* = 0.36 ^‡^	*p* = 0.49 ^‡^	*p* = 0.06 ^‡^
Fruit	16	5.75 ± 0.22 ^a^	5.06 ± 0.24 ^a^	4.63 ± 0.35 ^a^	5.44 ± 0.25 ^a^	2.94 ± 0.39 ^a^	1.25 ± 0.16 ^a^	5.63 ± 0.2 ^a^	4.06 ± 0.27 ^a^	4.63 ± 0.23 ^a^	5.19 ± 0.24 ^a^	1.25 ± 0.16 ^a^	5.63 ± 0.25 ^a^	5.63 ± 0.25 ^a^
Juice	16	5.56 ± 0.22 ^a^	5.00 ± 0.24 ^a^	5.13 ± 0.35 ^a^	5.38 ± 0.25 ^a^	2.81 ± 0.39 ^a^	1.44 ± 0.16 ^a^	5.06 ± 0.2 ^a^	3.94 ± 0.27 ^a^	4.38 ± 0.23 ^a^	4.63 ± 0.24 ^a^	1.50 ± 0.16 ^a^	5.38 ± 0.25 ^a^	4.94 ± 0.25 ^a^
Maturity		*p* ≤ 0.01 ^†^	*p* = 0.05 ^†^	*p* = 0.09 ^‡^	*p* ≤ 0.01 ^†^	*p* = 0.03 ^†^	*p* = 0.78 ^‡^	*p* ≤ 0.01 ^†^	*p* ≤ 0.01 ^†^	*p* ≤ 0.01 ^†^	*p* ≤ 0.01 ^†^	*p* = 1.00 ^‡^	*p* = 0.73 ^‡^	*p* = 0.01 ^†^
75%	16	4.75 ± 0.22 ^b^	4.68 ± 0.24 ^b^	4.44 ± 0.35 ^a^	4.88 ± 0.25 ^b^	3.50 ± 0.39 ^a^	1.38 ± 0.16 ^a^	4.81 ± 0.2 ^b^	4.88 ± 0.27 ^a^	3.69 ± 0.23 ^b^	4.25 ± 0.24 ^b^	1.38 ± 0.16 ^a^	5.44 ± 0.25 ^a^	4.81 ± 0.25 ^b^
100%	16	6.56 ± 0.22 ^a^	5.38 ± 0.24 ^a^	5.31 ± 0.35 ^a^	5.94 ± 0.25 ^a^	2.25 ± 0.39 ^b^	1.31 ± 0.16 ^a^	5.88 ± 0.2 ^a^	3.13 ± 0.27 ^b^	5.31 ± 0.23 ^a^	5.56 ± 0.24 ^a^	1.38 ± 0.16 ^a^	5.56 ± 0.25	5.75 ± 0.25 ^a^
Material by Maturity		*p* = 0.17 ^‡^	*p* = 0.85 ^‡^	*p* = 0.80 ^‡^	*p* = 0.85 ^‡^	*p* = 0.82 ^‡^	*p* = 0.78 ^‡^	*p* = 0.83 ^‡^	*p* = 0.75 ^‡^	*p* = 0.71 ^‡^	*p* = 0.36 ^‡^	*p* = 1.00 ^‡^	*p* = 0.09 ^‡^	*p* = 0.86 ^‡^
Fruit; 75%	8	4.63 ± 0.31 ^a^	4.75 ± 0.33 ^a^	4.13 ± 0.5 ^a^	4.88 ± 0.36 ^a^	3.63 ± 0.55 ^a^	1.25 ± 0.22 ^a^	5.13 ± 0.28 ^a^	5.00 ± 0.39 ^a^	3.88 ± 0.33 ^a^	4.38 ± 0.34 ^a^	1.25 ± 0.22 ^a^	5.88 ± 0.36 ^a^	5.13 ± 0.35 ^a^
Fruit; 100%	8	6.88 ± 0.31 ^a^	5.38 ± 0.33 ^a^	5.13 ± 0.5 ^a^	6.00 ± 0.36 ^a^	2.25 ± 0.55 ^a^	1.25 ± 0.22 ^a^	6.13 ± 0.28 ^a^	3.13 ± 0.39 ^a^	5.38 ± 0.33 ^a^	6.00 ± 0.34 ^a^	1.25 ± 0.22 ^a^	5.38 ± 0.36 ^a^	6.13 ± 0.35 ^a^
		*p* = 0.17 ^‡^	*p* = 0.85 ^‡^	*p* = 0.80 ^‡^	*p* = 0.85 ^‡^	*p* = 0.82 ^‡^	*p* = 0.78 ^‡^	*p* = 0.83 ^‡^	*p* = 0.75 ^‡^	*p* = 0.71 ^‡^	*p* = 0.36 ^‡^	*p* = 1.00 ^‡^	*p* = 0.09 ^‡^	*p* = 0.86 ^‡^
Juice; 75%	8	4.88 ± 0.31 ^a^	4.63 ± 0.33 ^a^	4.75 ± 0.5 ^a^	4.88 ± 0.36 ^a^	3.38 ± 0.55 ^a^	1.50 ± 0.22 ^a^	4.50 ± 0.28 ^a^	4.75 ± 0.39 ^a^	3.50 ± 0.33 ^a^	4.13 ± 0.34 ^a^	1.50 ± 0.22 ^a^	5.00 ± 0.36 ^a^	4.50 ± 0.35 ^a^
Juice; 100%	8	6.25 ± 0.31 ^a^	5.38 ± 0.33 ^a^	5.50 ± 0.5 ^a^	5.88 ± 0.36 ^a^	2.25 ± 0.55 ^a^	1.38 ± 0.22 ^a^	5.63 ± 0.28 ^a^	3.13 ± 0.39 ^a^	5.25 ± 0.33 ^a^	5.13 ± 0.34 ^a^	1.50 ± 0.22 ^a^	5.75 ± 0.36 ^a^	5.38 ± 0.35 ^a^
Average in samples	32	5.66 ± 0.15	5.03 ± 0.17	4.88 ± 0.25	5.41 ± 0.25	2.88 ± 0.27	1.34 ± 0.11	5.34 ± 0.14	4.00 ± 0.19	4.50 ± 0.17	4.91 ± 0.17	1.38 ± 0.11	5.50 ± 0.18	5.28 ± 0.18

Results are expressed as mean ± standard error. Values represented with different letters are statistically different at *p* ≤ 0.05; ^†^—significant factor in multifactor analysis; ^‡^—not significant factor in multifactor analysis. S—sensory evaluation; 1—intensity of red color; 2—flavor intensity; 3—flowery flavor; 4—fruity flavor; 5—green flavor; 6—off-flavor; 7—Taste; 8—acidic taste; 9— sweetness; 10—harmonious taste; 11—off-taste; 12—texture of sample firmness for fruits/homogeneity for juices; 13—overall sensory quality.

**Table 8:** Biologically active compounds in fresh strawberries, their juices, and by-products.

Parameters	*n*	TPC	ANT	HCA	FL
Material		*p* ≤ 0.01 ^†^	*p* ≤ 0.01 ^†^	*p* = 0.05 ^†^	*p* ≤ 0.01 ^†^
Fruit	4	58.19 ± 1.5 ^a^	26.75 ± 0.47 ^a^	14.51 ± 0.29 ^b^	2.92 ± 0.25 ^b^
Juice	4	35.27 ± 1.5 ^b^	22.08 ± 0.47 ^b^	9.06 ± 0.29 ^c^	0.55 ± 0.25 ^c^
By-Product	4	55.56 ± 1.5 ^a^	26.90 ± 0.47 ^a^	24.61 ± 0.29 ^a^	6.29 ± 0.25 ^a^
Maturity		*p* = 0.02 ^†^	*p* ≤ 0.01 ^†^	*p* ≤ 0.01 ^†^	*p* = 0.68 ^‡^
75%	6	52.51 ± 1.22 ^a^	23.45 ± 0.38 ^b^	16.43 ± 0.23 ^a^	3.23 ± 0.21 ^a^
100%	6	46.83 ± 1.22 ^b^	27.04 ± 0.38 ^a^	15.69 ± 0.23 ^b^	3.28 ± 0.21 ^a^
Material by Maturity		*p* = 0.07 ^‡^	*p* = 0.16 ^‡^	*p* = 0.01 ^†^	*p* = 0.07 ^‡^
Fruit; 75%	2	64.36 ± 2.39 ^a^	28.36 ± 1.04 ^a^	16.96 ± 0.38 ^a^	2.73 ± 0.36 ^a^
Fruit; 100%	2	52.02 ± 2.39 ^a^	25.13 ± 1.04 ^a^	12.06 ± 0.38 ^b^	3.12 ± 0.36 ^a^
		*p* = 0.09 ^‡^	*p* ≤ 0.01 ^†^	*p* ≤ 0.01 ^†^	*p* = 0.07 ^‡^
Juice; 75%	2	32.56 ± 1.23 ^a^	15.80 ± 0.31 ^b^	7.36 ± 0.20 ^b^	1.10 ± 0.36 ^a^
Juice; 100%	2	37.97 ± 1.23 ^a^	28.36 ± 0.31 ^a^	10.76 ± 0.20 ^a^	1.00 ± 0.36 ^a^
		*p* = 0.10 ^‡^	*p* = 0.11 ^‡^	*p* = 0.47 ^‡^	*p* = 0.07^‡^
By-product; 75%	2	60.62 ± 2.49 ^a^	26.19 ± 0.37 ^b^	24.96 ± 0.55 ^a^	6.87 ± 0.36 ^a^
By-product; 100%	2	50.51 ± 2.49 ^a^	27.62 ± 0.37 ^a^	24.26 ± 0.55 ^a^	5.71 ± 0.36 ^a^
Average in samples	12	49.67 ± 0.86	25.24 ± 0.27	16.06 ± 0.17	3.25 ± 0.15

Results are expressed as mean ± standard error. Values represented with different letters are statistically different at *p* ≤ 0.05; ^†^—significant factor in multifactor analysis; ^‡^—not significant factor in multifactor analysis. TPC—total phenolic compounds (mg GAE 100 mL^−1^); ANT—anthocyanins (mg Pg-3-G 100 mL^−1^); HCA—hydroxycinnamic acids (mg CAE 100 mL^−1^); FL—flavonols (mg QE 100 mL^−1^).

## Data Availability

Not applicable.

## References

[1] Basu A., Nguyen A., Betts N.M., Lyons T.J. (2013). Strawberry As a Functional Food: An Evidence-Based Review. Crit. Rev. Food Sci. Nutr..

[2] Afrin S., Gasparrini M., Forbes-Hernandez T.Y., Reboredo-Rodriguez P., Mezzetti B., Varela-López A., Giampieri F., Battino M. (2016). Promising health benefits of the strawberry: A focus on clinical studies. J. Agric. Food Chem..

[3] Giampieri F., Forbes-Hernandez T.Y., Gasparrini M., Alvarez-Suarez J.M., Afrin S., Bompadre S., Quiles J.L., Mezzetti B., Battino M. (2015). Strawberry as a health promoter: An evidence based review. Food Funct..

[4] Villamil-Galindo E., Van de Velde F., Piagentini A.M. (2021). Strawberry agro-industrial by-products as a source of bioactive compounds: Effect of cultivar on the phenolic profile and the antioxidant capacity. Bioresour. Bioprocess..

[5] Lorenzo J.M., Pateiro M., Domínguez R., Barba F.J., Putnik P., Kovačević D.B., Shpigelman A., Granato D., Franco D. (2018). Berries extracts as natural antioxidants in meat products: A review. Food Res. Int..

[6] Oszmiański J., Wojdyło A. (2008). Comparative study of phenolic content and antioxidant activity of strawberry puree, clear, and cloudy juices. Eur. Food Res. Technol..

[7] Skrovankova S., Sumczynski D., Mlcek J., Jurikova T., Sochor J. (2015). Bioactive compounds and antioxidant activity in different types of berries. Int. J. Mol. Sci..

[8] Aaby K., Skrede G., Wrolstad R.E. (2005). Phenolic composition and antioxidant activities in flesh and achenes of strawberries (*Fragaria ananassa*). J. Agric. Food Chem..

[9] Aaby K., Wrolstad R.E., Ekeberg D., Skrede G. (2007). Polyphenol composition and antioxidant activity in strawberry purees; Impact of achene level and storage. J. Agric. Food Chem..

[10] Hartmann A., Patz C.-D., Andlauer W., Dietrich H., Ludwig M. (2008). Influence of processing on quality parameters of strawberries. J. Agric. Food Chem..

[11] Fierascu R.C., Temocico G., Fierascu I., Ortan A., Babeanu N.E. (2020). Fragaria genus: Chemical composition and biological activities. Molecules.

[12] Azam M., Ejaz S., Naveed Ur Rehman R., Khan M., Qadri R. Postharvest Quality Management of Strawberries. In *Strawberry-Pre- and Post-Harvest Management Techniques for Higher Fruit Quality*. https://www.intechopen.com/books/6996.

[13] Barth E., Resende J.T.V.d., Moreira A.F.P., Mariguele K.H., Zeist A.R., Silva M.B., Stulzer G.C.G., Mafra J.G.M., Simões Azeredo Gonçalves L., Roberto S.R. (2020). Selection of experimental hybrids of strawberry using multivariate analysis. Agronomy.

[14] Šamec D., Maretić M., Lugarić I., Mešić A., Salopek-Sondi B., Duralija B. (2016). Assessment of the differences in the physical, chemical and phytochemical properties of four strawberry cultivars using principal component analysis. Food Chem..

[15] Granato D., Putnik P., Kovačević D.B., Santos J.S., Calado V., Rocha R.S., Cruz A.G.D., Jarvis B., Rodionova O.Y., Pomerantsev A. (2018). Trends in Chemometrics: Food Authentication, Microbiology, and Effects of Processing. Compr. Rev. Food Sci. Food Saf..

[16] Gündüz K., Özbay H. (2018). The effects of genotype and altitude of the growing location on physical, chemical, and phytochemical properties of strawberry. Turk. J. Agric. For..

[17] Aaby K., Mazur S., Nes A., Skrede G. (2012). Phenolic compounds in strawberry (*Fragaria x ananassa* Duch.) fruits: Composition in 27 cultivars and changes during ripening. Food Chem..

[18] Bursać Kovačević D., Putnik P., Dragović-Uzelac V., Vahčić N., Babojelić M.S., Levaj B. (2015). Influences of organically and conventionally grown strawberry cultivars on anthocyanins content and color in purees and low-sugar jams. Food Chem..

[19] Bursać Kovaćević D., Vahčić N., Levaj B., Dragović-Uzelac V. (2008). The effect of cultivar and cultivation on sensory profiles of fresh strawberries and their purées. Flavour Fragr. J..

[20] Oliver P., Cicerale S., Pang E., Keast R. (2018). Developing a strawberry lexicon to describe cultivars at two maturation stages. J. Sens. Stud..

[21] Kosar M., Kafkas E., Paydas S., Baser K.H.C. (2004). Phenolic composition of strawberry genotypes at different maturation stages. J. Agric. Food Chem..

[22] Hwang H., Kim Y.-J., Shin Y. (2019). Influence of ripening stage and cultivar on physicochemical properties, sugar and organic acid profiles, and antioxidant compositions of strawberries. Food Sci. Biotechnol..

[23] Paniagua C., Santiago-Doménech N., Kirby A.R., Gunning A.P., Morris V.J., Quesada M.A., Matas A.J., Mercado J.A. (2017). Structural changes in cell wall pectins during strawberry fruit development. Plant Physiol. Biochem..

[24] Bhat R., Geppert J., Funken E., Stamminger R. (2015). Consumers perceptions and preference for strawberries—A case study from Germany. Int. J. Fruit Sci..

[25] Lewers K.S., Newell M.J., Park E., Luo Y. (2020). Consumer preference and physiochemical analyses of fresh strawberries from ten cultivars. Int. J. Fruit Sci..

[26] Fan Z., Hasing T., Johnson T.S., Garner D.M., Barbey C.R., Colquhoun T.A., Sims C.A., Resende M.F.R., Whitaker V.M. (2021). Strawberry sweetness and consumer preference are enhanced by specific volatile compounds. Hortic. Res..

[27] Tchounwou P.B., Yedjou C.G., Patlolla A.K., Sutton D.J. (2012). Heavy Metal Toxicity and the Environment. Molecular, Clinical and Environmental Toxicology.

[28] Bekele Bahiru D., Yegrem L. (2021). Levels of Heavy Metal in Vegetable, Fruits and Cereals Crops in Ethiopia: A Review. Int. J. Environ. Monit. Anal..

[29] Parker C. (2014). Strawberry fields forever: Can consumers see pesticides and sustainability as an issue?. Sustain. Sci..

[30] Aktar W., Sengupta D., Chowdhury A. (2009). Impact of pesticides use in agriculture: Their benefits and hazards. Interdiscip. Toxicol..

[31] Abbasi H., Shah M.H., Mohiuddin M., Elshikh M.S., Hussain Z., Alkahtani J., Ullah W., Alwahibi M.S., Abbasi A.M. (2020). Quantification of heavy metals and health risk assessment in processed fruits’ products. Arab. J. Chem..

[32] Tiwari B.K., Muthukumarappan K., O’Donnell C.P., Cullen P.J. (2008). Colour degradation and quality parameters of sonicated orange juice using response surface methodology. LWT-Food Sci. Technol..

[33] Bursać D., Vahčić N., Levaj B., Dragović-Uzelac V., Biško A. (2007). The influence of cultivar on sensory profiles of fresh and processed strawberry fruits grown in Croatia. Flavour Fragr. J..

[34] Bursać Kovačević D., Putnik P., Dragović-Uzelac V., Pedisić S., Režek Jambrak A., Herceg Z. (2016). Effects of cold atmospheric gas phase plasma on anthocyanins and color in pomegranate juice. Food Chem..

[35] Škegro M., Putnik P., Bursać Kovačević D., Kovač A.P., Salkić L., Čanak I., Frece J., Zavadlav S., Ježek D. (2021). Chemometric Comparison of High-Pressure Processing and Thermal Pasteurization: The Nutritive, Sensory, and Microbial Quality of Smoothies. Foods.

[36] Howard L.R., Clark J.R., Brownmiller C. (2003). Antioxidant capacity and phenolic content in blueberries as affected by genotype and growing season. J. Sci. Food Agric..

[37] Lee J., Durst R.W., Wrolstad R.E. (2005). Determination of total monomeric anthocyanin pigment content of fruit juices, beverages, natural colorants, and wines by the pH differential method: Collaborative study. J. AOAC Int..

[38] Kelly K., Whitaker V.M., Nunes M.C.d.N. (2016). Physicochemical characterization and postharvest performance of the new Sensation^®^ ‘Florida127′ strawberry compared to commercial standards. Sci. Hortic..

[39] De Jesús Ornelas-Paz J., Yahia E.M., Ramírez-Bustamante N., Pérez-Martínez J.D., del Pilar Escalante-Minakata M., Ibarra-Junquera V., Acosta-Muñiz C., Guerrero-Prieto V., Ochoa-Reyes E. (2013). Physical attributes and chemical composition of organic strawberry fruit (*Fragaria x ananassa* Duch, cv. Albion) at six stages of ripening. Food Chem..

[40] Nunes M.C.N., Brecht J.K., Morais A.M.M.B., Sargent S.A. (2006). Physicochemical changes during strawberry development in the field compared with those that occur in harvested fruit during storage. J. Sci. Food Agric..

[41] Olsson M.E., Ekvall J., Gustavsson K.-E., Nilsson J., Pillai D., Sjöholm I., Svensson U., Åkesson B., Nyman M.G.L. (2004). Antioxidants, low molecular weight carbohydrates, and total antioxidant capacity in strawberries (*Fragaria x ananassa*):  Effects of cultivar, ripening, and storage. J. Agric. Food Chem..

[42] Concha--Meyer A.A., D’Ignoti V., Saez B., Diaz R.I., Torres C.A. (2016). Effect of storage on the physico--chemical and antioxidant properties of strawberry and kiwi leathers. J. Food Sci..

[43] Belakud B., Bahadur V., Prasad V.M. (2015). Performance of strawberry (*Fragaria x ananassa* Duch.) varieties for yield and biochemical parameters. Pharma Innov. J..

[44] Shao W.-C., Zang Y.-Y., Ma H.-Y., Ling Y.E., Kai Z.-P. (2021). Concentrations and related health risk assessment of pesticides, phthalates, and heavy metals in strawberries from Shanghai, China. J. Food Prot..

[45] Bystricka J., Musilova J., Trebichalsky P., Tomas J., Stanovic R., Bajcan D., Kavalcova P. (2015). The relationships between content of heavy metals in soil and in strawberries. Int. J. Phytoremediation.

[46] Elbagermi M.A., Edwards H.G.M., Alajtal A.I. (2012). Monitoring of Heavy Metal Content in Fruits And Vegetables Collected from Production and Market Sites in the Misurata Area of Libya. ISRN Anal. Chem..

[47] Official Journal of the European Union, L 364, 20 December 2006. https://eur-lex.europa.eu/legal-content/EN/TXT/?uri=OJ:L:2006:364:TOC.

[48] Commission Regulation (EU) 2021/1317. https://eur-lex.europa.eu/eli/reg/2021/1317/oj.

[49] Commission Regulation (EU) 2021/1323. https://eur-lex.europa.eu/legal-content/EN/TXT/?uri=CELEX%3A32021R1323.

[50] EFSA Panel on Contaminants in the Food Chain (CONTAM) (2010). Scientific Opinion on Lead in Food. EFSA J..

[51] Fernández-Ortuño D., Chen F., Schnabel G. (2012). Resistance to Pyraclostrobin and Boscalid in *Botrytis cinerea* Isolates from Strawberry fields in the Carolinas. Plant Dis..

[52] Krieger R. (2010). Hayes’ Handbook of Pesticide Toxicology.

[53] https://www.fao.org/fileadmin/user_upload/IPM_Pesticide/JMPR/Evaluations/2007/Pyrimethanil.pdf.

[54] https://pubchem.ncbi.nlm.nih.gov/compound/Pyrimethanil.

[55] Baggio J.S., Peres N.A., Amorim L. (2018). Sensitivity of Botrytis cinerea Isolates from Conventional and Organic Strawberry Fields in Brazil to Azoxystrobin, Iprodione, Pyrimethanil, and Thiophanate-Methyl. Plant Dis..

[56] Faniband M., Ekman E., Littorin M., Maxe M., Larsson E., Lindh C.H. (2019). Biomarkers of Exposure to Pyrimethanil After Controlled Human Experiments. J. Anal. Toxicol..

[57] Weber F., Larsen L.R. (2017). Influence of fruit juice processing on anthocyanin stability. Food Res. Int..

[58] Orton F., Rosivatz E., Scholze M., Kortenkamp A. (2011). Widely Used Pesticides with Previously Unknown Endocrine Activity Revealed asin VitroAntiandrogens. Environ. Health Perspect..

[59] Gupta P.K., Gupta R.C. (2018). Toxicity of Fungicides. Veterinary Toxicology.

[60] Waechter F., Weber E., Hertner T. (2001). Cyprodinil: A Fungicide of the Anilinopyrimidine Class. Handbook of Pesticide Toxicology.

[61] Seeland A., Oehlmann J., Müller R. (2012). Aquatic ecotoxicity of the fungicide pyrimethanil: Effect profile under optimal and thermal stress conditions. Environ. Pollut..

[62] Malhat F.M., Loutfy N.M., Thabet W. (2014). Dissipation Profile and Human Risk Assessment of Pyrimethanil Residues in Cucumbers and Strawberries. J. Health Pollut..

[63] Aprea E., Biasioli F., Carlin S., Endrizzi I., Gasperi F. (2009). Investigation of volatile compounds in two raspberry cultivars by two headspace techniques: Solid-phase microextraction/gas chromatography−mass spectrometry (SPME/GC−MS) and proton-transfer reaction−mass spectrometry (PTR−MS). J. Agric. Food Chem..

[64] Lu H., Ban Z., Wang K., Li D., Li D., Poverenov E., Li L., Luo Z. (2017). Aroma volatiles, sensory and chemical attributes of strawberry (*Fragaria x ananassa* Duch.) achenes and receptacle. Int. J. Food Sci. Technol..

[65] Aubert C., Bruaut M., Chalot G., Cottet V. (2020). Impact of maturity stage at harvest on the main physicochemical characteristics, the levels of vitamin C, polyphenols and volatiles and the sensory quality of Gariguette strawberry. Eur. Food Res. Technol..

[66] Wei M., Wang H., Ma T., Ge Q., Fang Y., Sun X. (2021). Comprehensive utilization of thinned unripe fruits from horticultural crops. Foods.

[67] Jančářová I., Jančář L., Náplavová A., Kubáň V. (2013). Changes of organic acids and phenolic compounds contents in grapevine berries during their ripening. Open Chem..

[68] Jiao Y., Chen D., Fan M., Young Quek S. (2019). UPLC-QqQ-MS/MS-based phenolic quantification and antioxidant activity assessment for thinned young kiwifruits. Food Chem..

[69] Zheng H.-Z., Kim Y.-I., Chung S.-K. (2012). A profile of physicochemical and antioxidant changes during fruit growth for the utilisation of unripe apples. Food Chem..

[70] Labbé M., Ulloa P.A., López F., Sáenz C., Peña Á., Salazar F.N. (2016). Characterization of chemical compositions and bioactive compounds in juices from pomegranates (Wonderful, Chaca and Codpa) at different maturity stages. Chil. J. Agric. Res..

[71] Heng Koh T., Melton L.D. (2002). Ripening-related changes in cell wall polysaccharides of strawberry cortical and pith tissues. Postharvest Biol. Technol..

[72] Siebeneichler T.J., Crizel R.L., Camozatto G.H., Paim B.T., da Silva Messias R., Rombaldi C.V., Galli V. (2020). The postharvest ripening of strawberry fruits induced by abscisic acid and sucrose differs from their in vivo ripening. Food Chem..

[73] Haile Z.M., Nagpala-De Guzman E.G., Moretto M., Sonego P., Engelen K., Zoli L., Moser C., Baraldi E. (2019). Transcriptome Profiles of Strawberry (Fragaria vesca) Fruit Interacting With Botrytis cinerea at Different Ripening Stages. Front. Plant Sci..

